# Risk factors for autism spectrum disorder among infants and children admitted in a neonatal intensive care unit

**DOI:** 10.1016/j.dialog.2026.100288

**Published:** 2026-03-19

**Authors:** Marwa Magdy Mohamed, Safaa AbdELHamid ELMeneza, Safaa Mahmoud Hammouda

**Affiliations:** a Department, Faculty of Medicine for Girls, Al-Azhar University, Egypt; b Neonatology Department, Faculty of Medicine for Girls, Al-Azhar University, Egypt; c Faculty of Medicine for Girls, Al-Azhar University, Egypt

**Keywords:** Autism spectrum disorder (ASD), High-risk infants, NICU, Auditory brainstem response (ABR), CARS-2-St-test, Newborn infants

## Abstract

**Background and objective:**

Autism Spectrum Disorder (ASD) is a diverse neurodevelopmental condition. This study aimed to highlight the prevalence and risk factors associated with the development of ASD in infants and children who have been admitted to the NICU.

**Methods:**

A cross-sectional study was conducted on 66 pediatric outpatient clinic infants and children <3 years of age with a history of admission to NICU. CARS-2St questionnaire and Auditory brain response (ABR) were used.

**Results:**

The percentage of ASD was 9.1% (6/66) and the mean total CARS-2-St-test score of ASD vs non-ASD cases was 35 ± 2.7 vs 18.58 ± 2.74, *p* = 0.001. Significant risk factors for ASD included premature rupture of membranes (p = 0.001), prematurity (*p* = 0.002), longer duration of admission (*p* = 0.038), and increased need for respiratory support (*p* = 0.034). The mean values of the absolute latencies of ABR waves III, IV, and V and the I-III and I-V intervals of the right and left ear were significantly increased in those with ASD.

**Conclusions:**

This study is exploratory and hypothesis-generating and highlights the importance of screening infants and children with a history of admission to NICU who have an increased risk of ASD, and should be screened even if they have no other neurological manifestations.

## Highlights

• The study identified maternal, neonatal, and environmental risk factors for ASD in NICU graduates.
• Future research on ABR as a potential early identification tool for ASD is suggested.
• Even infants without apparent neurological abnormalities may still develop ASD. What is known and what is new?
• Known: - ASD is a heterogeneous neurodevelopmental disorder. - Cultural challenges and the level of awareness hinder diagnosis/treatment in developing countries. - Risk factors for ASD are not fully understood.
• New: - The study identified specific maternal, neonatal, and environmental risk factors. - Proposed ASD screening for NICU-admitted infants/children. - Suggested ABR future research as a potential early identification tool for ASD. What is the implication, and what should change now?
• Implications: Early screening and intervention are critical for NICU graduates, even if no overt neurological issues.
• Changes Needed: - Implement routine ASD screening for NICU graduates. - Further explore ABR's role in ASD risk assessment. - Address cultural barriers to improve ASD diagnosis and care in developing regions.

## 1 Introduction

### 1.1 Background

#### 1.1.1 Biologic and medical factors

Autism Spectrum Disorder (ASD) is a neurodevelopmental condition characterized by difficulties in social engagement and the presence of limited interests and repetitive behaviors. Concerns are mounting over its rising prevalence [1]. The social communication and interaction as well as restricted and repetitive behaviors. The social communication and interaction include difficulty in social-emotional reciprocity; difficulty with the “back-and-forth” of social interaction, such as sharing interests, emotions, or sustaining a standard conversation; nonverbal communication embracing differences in using or understanding eye contact, facial expressions, gestures, and body language and relationship management; challenges in developing, maintaining, and understanding relationships, which may include difficulty adjusting behavior to suit different social contexts. The restricted and repetitive behaviors incorporating repetitive movements or speech; such as hand-flapping, or repeating phrases, insistence on sameness; a strong preference for rigid routines and extreme distress caused by minor changes, highly restricted interests; intense, focused interests in specific topics or objects that are unusual in their depth or nature and sensory processing differences; hyper- or hypo-reactivity to sensory input, such as being overwhelmed by loud noises or lights, or having an unusual fascination with certain textures or smells [2]. ASD includes different disorders, which are distinguished by various degrees of complexity in social contact and communication. Other features include abnormal forms or arrays of actions and behaviors, for instance, difficulty switching between activities, concentration on details, and atypical responses to sensations. According to Zeidan et al. [3],1/100 children have autism, but the prevalence of autism in low- and middle-income countries has not been well-studied. In the USA, it was observed that the prevalence in 8-Year-Olds was 32.2 per 1000 children, which is equivalent to approximately 1 in 31 children [4].

Early management, during the first two years of life, may improve prognosis [5]. However, ASD is not recognized till 2 to 5 years of age [5]. This delay in diagnosis may be due to a lack of awareness of the early symptoms of ASD, and/or limited access to a specialist, which delays the diagnosis and subsequent management.

Evolving evidence proposes an association between admission to the NICU and diagnosis of ASD [6]. High-risk neonates are subjected to several clinical maneuvers related to painful stimuli and stress, as well as adverse environmental conditions such as noise and light. Maternal, intrauterine, and early postnatal environments permanently alter the neonatal brain [6], [7], [8].

#### 1.1.2 Environmental challenges

There is evidence that emphasizes maternal metabolic, nutritional, immunologic, psychiatric, nutritional, and toxic exposures for ASD. Also, postnatal complications that interact with prenatal vulnerabilities has important role. Metabolic risk factors include maternal obesity, gestational diabetes, and infections during pregnancy, have been repeatedly identified as significant ASD risk factors [9]. Maternal depression, fever, and a broad range of psychiatric disorders have been linked to increased ASD risk. [10] Psychosocial stress, occupational exposures, and environmental injustice may compound risk in marginalized and low income populations [11] Maternal exposure to antibiotics, SSRIs, smoking, household pesticides, heavy metals (e.g., arsenic, cadmium, lead), and agricultural pesticides has shown consistent association with elevated ASD risk. Exposure to environmental toxicants during gestation such as solvents, methanol, organohalogens, and indoor pesticides, directly affects fetal neural development [12].

Early postnatal factors have also been linked to ASD. Complications at delivery and neonatal problems such as newborn metabolic disturbances or perinatal stress are associated with increased ASD risk [12]. Early life exposure to toxic substances, including some environmental pollutants, may further influence neurodevelopment after birth. [13].Postnatal effects may be especially significant in infants already genetically or prenatally predisposed to ASD traits.

#### 1.1.3 Social and cultural challenges

Beyond biologic and clinical contributors, social and cultural challenges significantly affect the diagnosis and management of ASD. In many developing countries, limited awareness of early symptoms delays recognition and intervention. There are cultural challenges to the diagnosis and treatment of ASDs, besides a low awareness in developing countries. Cultural beliefs may lead to underreporting of concerns or misinterpretation of behaviors, while societal stigma can discourage families from seeking evaluation [13].

The social and financial burdens begin as families adjust to meeting the needs of a child with autism, while differences in neurodevelopment and learning further contribute to challenges in social interaction, as well as ongoing difficulties in behavior and communication [14]. Insufficient access to prompt ASD screening assessments postpones the start of interventions that enhance developmental outcomes [15].

### 1.2 Rationale and knowledge gap

There is risk of development of ASD among neonates admitted to the NICU [16]. Additionally, there is insufficient information about the risk factors involved, particularly when motor system issues or cerebral palsy are not present [16]. Recognizing the scope of the risks of ASD development in early infancy could facilitate early diagnosis and treatment, and thereby enhance the quality of life.

### 1.3 Aim

This study aimed to highlight the prevalence and risk factors associated with the development of ASD in infants and children admitted to the NICU in a developing Country.

## 2 Methods

### 2.1 Patient and data

This study was approved by ethics committee at Faculty of Medicine for Girls, Al-Azhar University and the ethics approval was ID 1639, number 2022121639.This cross-sectional study was conducted at the pediatric outpatient clinic of the Health Insurance Hospital in Kafr El Sheikh Governorate from July 2023 to March 2024. The parents of infants and children who visited the outpatient clinic were asked if they had a history of admission after birth to the NICU. We estimated the minimum sample size calculation for 64 infants and children required for the study based on a power of 80% and 2-side significance level of 0.05 using the reported risks of ASD of 2.8% in the general population and 10% in infants and children following NICU admission [17], [18].

Infants and children up to the third year of age with a history of NICU admission were included in the study.

Cases with proven metabolic or congenital anomalies, complex congenital heart disease (structural abnormalities that often requires multiple surgical procedures for correction), cerebral palsy/motor system abnormalities, or incomplete, inaccurate medical history were excluded.

### 2.2 Procedures

A thorough history was taken from each case of perinatal, natal, and postnatal indications for NICU admission as shown in Supplement 1. All cases had full clinical examinations, neurological evaluation, followed by administration of the CARS - 2-ST questionnaire to the parents by the pediatrician and psychiatrist. CARS-2-ST is a screening instrument. This was followed by an auditory brainstem response (ABR) examination.

### 2.3 The psychological test (CARS - 2-ST) questionnaire

The CARS-2-ST was employed as described by Schopler, Reichler, and Renner in 1988 [19] and Alqahtani in 2020 [20]. The response to each question was scored between 1 and 4, 1 indicating normal for the child's age, 2 mildly abnormal, 3 moderately abnormal, and 4 severely abnormal. CARS-2-S*T* test comprises a 15-item behavioral rating scale designed to differentiate autism from other developmental disorders and assess the severity of autism [19], [21]. It is renowned for its high reliability, validity, and inter-rater agreement, and applies to children of all ages, including preschoolers [21]. Scores ranging from 30 to 36.5 suggest mild to moderate autism, whereas scores from 37 to 60 indicate severe autism. Scores below 30 are considered non-autistic [19], [22]. To minimize bias, the questions were explained to the parents before the test.

### 2.4 ABR examination

ABR was performed using Eclipse Ep25 (Interacoustics Assens, Denmark). It is a commercial device from Interacoustics in Assens, Denmark, to evaluate auditory pathways in an objective, non-invasive manner by recording brainstem electrical activity in response to sound stimuli. ABR is a test that assesses the functional integrity of the auditory system, from the inner ear (cochlea) to the brainstem. The electrodes were placed on the head to pick up electrical activity. Stimuli were applied as clicks [23]. The recording and analysis of the electrical activity that occurred in response to these sounds was performed by the system to identify peaks and measure various waves of the auditory brainstem response.

ABR waves (I–V) reflect sequential activation of neuroanatomical structures along the auditory nerve and brainstem, beginning with the distal and proximal auditory nerve (Waves I–II), through the cochlear nucleus and superior olivary complex (Wave III), and extending to the lateral lemniscus and inferior colliculus in the midbrain (Waves IV–V). Alterations in ABR wave latency or morphology, therefore, suggest functional differences within these brainstem auditory circuits. Disruptions in these pathways are increasingly recognized as part of ASD-related neuropathology [24].

### 2.5 Statistical analysis

The data were summarized using mean ± standard deviation (± SD) or median ± interquartile range as appropriate, and frequencies and percentages, as appropriate. Numerical variables were compared between study groups using *t*-test for normally distributed data and Mann-Whitney *U* test for non-normally distributed. Chi-square (χ^2^) test, or Fisher's exact test when cell counts were less than 5, was used to compare categorical data. A two-sided *p*-value of 0.05 or less was deemed statistically significant. Multivariable logistic regression analysis was used to explore the 0interaction between the variables which showed significance in univariate analysis.

All statistical analyses were performed using IBM SPSS Statistics for Windows, version 25 (IBM Corp, Armonk, NY, USA).

## 3 Results

### 3.1 Characteristics of the studied groups

**Fig. 1:**
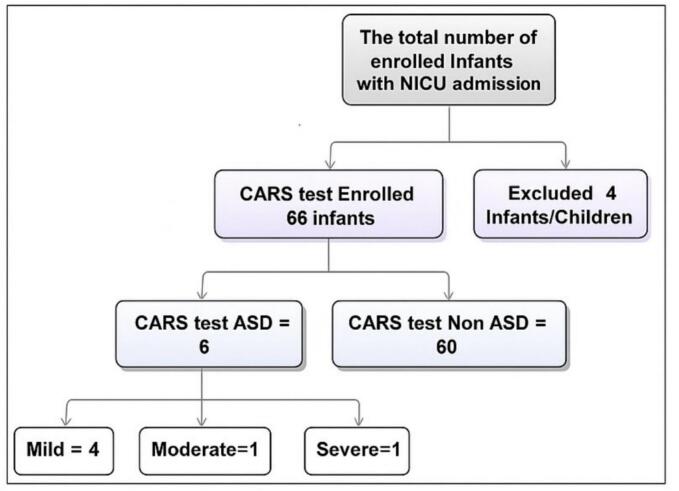
Study flow diagram.

The total number of patients who attended the clinic during the study period was 3360. We initially screened 70 infants and children with a history of NICU admission after birth, but only 66 (94.2%) met the inclusion criteria. Six (9.1%) of the 66 had ASD. Fig. 1

There was a significantly lower mean gestational age among the autistic infants and children, median and IQR 32 (38–31) vs 37 (37–37) weeks (*p* = 0.032). Supplement 2.

### 3.2 Autistic rating scale (CARS)

All 15 items had significantly higher median scores among the autistic cases (Table 1). Four children (66%) had mild scores, while one child (16%) each had moderate and severe scores. The mean score for the ASD group was 35 ± 2.7 vs 18.58 ± 2.74 for the control group (*P* = 0.001). Table 1.

**Table 1:** Comparison between autistic and non-autistic cases according to the childhood autism rating scale (CARS).

CARS Questions		Non- autistic Median (IQR)	Autistic Median (IQR)	*p* value
Relations to people		1(1–2)	3(2.375–3)	<0.001
Imitation		1(1–1)	2(1.875–2.75)	<0.001
Emotional response		1(1–1)	1(2–3.25)	<0.001
Body use		1(1–1)	2(2–2.625)	<0.001
Object use		1(1–2)	2.5(1.875–3)	0.004
Adaptation to change		1(1–2)	2.25(1.75–3)	0.018
Visual response		2(1–2)	3(2.75–3)	<0.001
Listening response		1(1–2)	2.5(2.375–3)	0.001
Taste, smell, touch		1(1–1)	2(2–3)	0.001
Fear or nervousness		1(1–1)	2(1.75–2.125)	0.004
Verbal communication		1(1–1)	2.25(2–3)	<0.001
Activity level		1(1–1.75)	2(1.875–2)	0.006
Nonverbal communication		1(1–1)	2(2–3)	<0.001
Level and consistency of intellectual response		1(1–1)	2(1.375–2.25)	0.006
General impression		1(1–1)	2(2–3)	<0.001
Total score	Mean ± SD	18.58 ± 2.74	35 ± 2.7	<0.001
Total ASD = 6	N		%	
Mild	4		66.6%	
Moderate	1		16.7%	
Severe	1		16.7%	

Mann-Whitney U test Independent T-test.

### 3.3 Prenatal –natal and post-natal risk factors

The differences between children with and without ASD are shown in Table 2 and Supplements 2 and 3. The significant differences included a higher rate of premature rupture of membranes. (PROM) (P = 0.001), prematurity (*P* = 0.002), need for respiratory support (*P* = 0.03), and use of mechanical ventilator (*P* = 0.02) or CPAP (*p* = 0.007) among children with ASD. Table 2.

**Table 2:** Risk factors among the studied groups.

Risk Factors			Non- autistic N (60) (%)	Autistic آN(6) (%)	*P* value
Premature Rupture of Membranes	No		59(98.3)	3(50)	0.001
	Yes		1(1.7)	3(50)	
Prematurity	No		55 (91.7)	2(33.3)	0.002
	Yes		5(8.3)	4(66.7)	
Respiratory support	No		47(78.3)	2(33.3)	0.034
	Yes		13(21.7)	4(66.7)	
Type of respiratory support	Mechanical ventilator	No	55(91.7)	3(50)	0.02
		Yes	5(8.3)	3(50)	
	Nasal oxygen	No	50(83.3)	3(50)	0.08
		Yes	10(16.7)	3(50)	
	Continuous positive airway pressure	No	60(100)	4(66.7)	0.007
		Yes	0(0)	2(33.3)	
Duration of oxygen support			Median (IQR)		
			15(0–20.25)		
Duration of NICU admission			Median (IQR)		
			23(6.75–30)		

Fisher's exact test Mann-Whitney U test.

### 3.4 ABR

There were significant increases in the latencies of waves lll, IV, and V, and in I-III and I-V intervals in both the right and left ears in autistic infants and children with ASD, as shown in Table 3. However, a comparison of the right and left ear wave latencies and interaural amplitude differences (IAD) showed no statistically significant differences between the right and left ears of those with ASD, as shown in supplement 3. The auditory brainstem pathways on the right and left sides appear to function similarly in most respects. This suggests that ASD related ABR abnormalities when present are generally bilateral/systemic, rather than affecting only one ear. A significant right–left asymmetry in wave V latency or amplitude suggests possible differences in how each hemisphere processes later stages of auditory information.

**Table 3:** Comparison between the auditory brainstem response (ABR) of the right ear and left ear in autistic cases.

Wave	Autistic right ear	Autistic left ear	P value
(ms)	(Mean ± SD)	(Mean ± SD)	
Wave I	1.73 ± 0.1	1.9 ± 0.4	0.336
Wave II	2.8 ± 0.16	3.2 ± 0.72	0.214
Wave III	4.87 ± 0.4	5.2 ± 0.5	0.232
Wave IV	5.58 ± 0.24	6 ± 0.4	0.052
Wave V	6.7 ± 0.24	7 ± 0.2	0.0405
I-III interval	3.13 ± 0.42	3.3 ± 0.6	0.58
III-V	1.88 ± 0.39	1.8 ± 0.35	0.716
I-V	5 ± 0.33	5.1 ± 0.3	0.59

Independent T test.

### 3.5 Risk factors for ASD

Exploratory univariate analyses showed that the duration of NICU admission, prematurity, gestational age, weight, need for respiratory support, duration of oxygen support, and PROM were the significant risk factors for ASD, but none of them attained significance on multivariable regression analysis Table 4.

**Table 4:** Univariate and multivariable logistic regression to detect significant predictors of being autistic.

Predictors	Univariate regression			Multivariable regression		
	Unadjusted odds ratio	95% CI	P value	Adjusted odds ratio	95% CI	P value
Duration of NICU admission	1.09	1.02–1.19	0.037	0.674	0.04–1.13	0.137
Prematurity	0.045	0.005–0.423	0.007	0.00	0.00–3.47	0.073
Gestational age	0.728	0.541–0.978	0.035			
Weight	0.732	0.556–0.965	0.027			
Respiratory support	0.132	0.018–0.936	0.043			
Duration of O2 therapy	1.16	1.033–1.305	0.012			
Premature rupture of membranes	0.043	0.003–0.564	0.016			
Breastfeeding	0.667	0.047–9.472	0.765			
Formula feed	1.5	0.116–19.44	0.756			

CI confidence interval.

Univariate regression analysis of ABR variables showed a significant association of right wave lll and V latencies and RT l-V interval with the diagnosis of ASD (Supplement 5). There was also a similar association regarding the left, as shown in Supplement 4. The small ASD group likely constrained the multivariable analysis; one should not over-interpret odds ratios derived from very small cell sizes.

## 4 Discussion

This study is exploratory and hypothesis-generating. It was surprising that none of the parents had been told before by healthcare providers that their child had ASD and had not been undergone to ASD tests. This indicates a lack of awareness of early ASD symptoms that may contribute to delayed diagnosis. Our findings suggest that incorporating ASD screening tools into neonatal follow-up programs could be beneficial, but further research with larger samples is needed to explore this possibility.

All 15 items in the CARS test were significantly associated with an increased risk of ASD. Each item assesses a specific behavior commonly associated with ASD, such as imitation, emotional response, and verbal communication. A higher score on any of these items indicates a greater severity of autism-related behavior. The higher scores correlate with greater ASD symptom severity.

This cross-sectional study showed that 9% of infants and children with a history of NICU admission have ASD. An earlier report showed that 10% to 25% of all ASD cases are likely to be NICU graduates [25]. The observation that ASD appears more prevalent in infants born at earlier gestational ages such as 32 weeks compared with 37 weeks is highly compelling and strongly supported by current epidemiologic data. A Swedish national cohort of over four million births showed ASD prevalence rates of 2.6% in very to moderate preterm infants (28–33 weeks) compared with 1.6% in early term and 1.4% in full term infants, underscoring a clear gradient of increasing ASD risk with decreasing gestational age [26]. Moreover, meta-analytic data further reinforce this pattern, indicating that preterm birth elevates the likelihood of ASD by approximately 3.3-fold relative to the general population. Given these findings, the current results pattern of higher ASD prevalence in younger EGA infants is biologically plausible and aligns with established literature. Evidence is linking prematurity, neuroinflammatory vulnerability, and altered neurodevelopment to increased ASD [18].

This study showed a significant correlation between the risk of ASD and PROM but not maternal age, chronic diseases, fever, and mode of delivery. Our results agree with those from other studies [27], [28], [29], [30]. PROM triggers lowering of the neonate's insulin-like factor-1 that aids in neoneuronal myelination and is associated with elevated levels of IL6 in the cerebellum of many autistic children [28], [29], [30]. The results of our study also further support the role of prematurity in the development of ASD [18], [31], [32].

The vulnerability of preterm infants to ASD is strongly linked to perinatal neuroinflammatory processes. Preterm birth often results from or is accompanied by inflammatory conditions, including preterm premature rupture of membranes (pPROM), where the loss of fetal membrane integrity exposes the fetus to infection-related cytokines, oxidative stress, and microbial byproducts that can disrupt neurodevelopmental trajectories. Neuroinflammation, particularly microglial activation during critical periods of brain maturation, impairs synaptogenesis and white matter development, mechanisms central to ASD pathophysiology and frequently observed in preterm infants.

Subsequent elevation of pro-inflammatory cytokines such as IL 1β, IL 6, and TNF α can cross or disrupt the blood–brain barrier. These cytokine-driven insults may disrupt connectivity pathways and impair synaptic refinement, consistent with observed ASD phenotypes in preterm populations, where neuroinflammation and microglial dysregulation are recognized contributors to atypical brain development [33], [34].

Although the results did not attain significance on multivariable regression analysis due to the small size of the ASD group, it is important to note that only prematurity attained a measure of independence as a risk factor for ASD. Preterm newborns frequently undergo extended pain-inducing procedures during their stay in NICU [35], and these have been lined to changes in stress hormone levels, which influence “emotion and cognition habits [36], [37].

About two-thirds of cases of ASD in this study were males. The higher proportion of males is in accordance with the results of earlier studies [20] with the exception of a few who reported a higher proportion of females among children with ASD who were born from 25 to 27 weeks of gestation [37], [38]. The association between the increased development of ASD and postnatal circumstances, such as the need for respiratory support, including mechanical ventilators and CPAP, history of cyanosis, and increased length of stay in the NICU, is also in accordance with the results of previous studies [39], [40].

The finding of significant prolongation of absolute latencies of ABR waves (I, III, and V) in both ears of children with ASD is like the findings in other studies [41]. These findings could reflect damage to the brainstem in these patients [42], [43], [44], [45]. The differences in inter-peak latencies (IPL) between ASD and non-ASD groups might also be indicative of auditory nerve and cochlear pathology [44], [45]. These findings taken together suggest that the ABR response may be helpful in the diagnosis of ASD. These findings aim to inform ongoing efforts and guide future research directions. The ABR abnormalities observed in individuals with ASD may reflect underlying neurodevelopmental differences within the auditory pathway, particularly those related to brainstem circuit maturation and synaptic transmission. Autistic children had prolonged ABR wave latencies, reduced amplitudes, or atypical inter-peak intervals, indicating slower or less efficient neural conduction through the auditory brainstem. These findings are thought to reflect disruptions in early neurodevelopmental processes such as altered excitatory inhibitory balance and synaptic signaling, differences in maturation of myelinated auditory pathways, and structural and functional changes in auditory brainstem nuclei. A subset of autistic children shows distinct speech-evoked ABR patterns associated with language-related cortical differences, supporting the concept of a neurophysiological ASD subtype related to auditory brainstem processing. Although ABR abnormalities are statistically more common in ASD than in typically developing children, current evidence does not support their use as a diagnostic or screening tool. Its clinical role remains limited to providing complementary information alongside behavioral, developmental, and neurological assessments [24], [46], [47].

### 4.1 Strengths and limitations

Our study has several strengths. Among these is that it is one of the few with a focus on the prevalence of ASD among infants and children who have a history of NICU admission the non-western population, whereas other studies [23], [28], [34] looked at ASD in the general population. Also, we included younger-age infants and those with no motor neurological disabilities. In addition, our results provide support for the potential use of ABR in the early identification of ASD. ABR may be associated with the identification of ASD.

However, our study also has several limitations; it was limited to one hospital and clinic –based sampling, so results may not be adequately generalized, and it did not explore any association between socioeconomic factors and ASD that may affect the culture and parent's behavior and awareness of ASD. Also, the study did not include other infants and children who were not admitted to the NICU, which affects the comparison of risk factors for ASD in the general population. Other limitation is reliance on a single screening instrument rather than diagnostic confirmation. The small ASD group likely constrained the multivariable analysis. ABR findings in premature infants, although not previously described in the ASD literature, must be interpreted through the well-established body of research on developmental delay, brainstem maturation, and prematurity.

### 4.2 Implications and actions needed

Our results suggest the need for large cohort studies and a focus on the need for interventions after discharge from the NICU. The results also indicate the need for prospective early screening of such infants and children for ASD.

Future research is needed to confirm its prospective role. The finding of significant prolongation of absolute latencies of ABR waves (I, III, and V) in both ears of children with ASD and inter-peak latencies between ASD and non-ASD groups showed the altered auditory processing of the ASD participants, still need further researches with larger sample size to distinguish between physiological differences and clinical utility, as well as to provide sensitivity, and predictive values.

## 5 Conclusions

The percentage of ASD identified in this clinic-based cohort is 9.1% among infants and children with a history of NICU admission. It is notably high, particularly given that these children were otherwise clinically neurologically normal. While this study was exploratory and hypothesis generating, it revealed that prematurity may be an important risk factor for ASD. In addition, preliminary data suggest that periodic assessment of neonates admitted to NICU is important for early identification of ASD

## Reporting checklist

The authors have completed the STROBE reporting checklist.

## CRediT authorship contribution statement

**Marwa Magdy Mohamed:** Writing – original draft, Resources, Data curation, Conceptualization. **Safaa AbdELHamid ELMeneza:** Writing – review & editing, Supervision, Project administration, Funding acquisition, Formal analysis. **Safaa Mahmoud Hammouda:** Writing – review & editing, Writing – original draft, Visualization, Validation, Software, Resources, Methodology, Investigation.

## Ethical statement

The authors are accountable for all aspects of the work in ensuring that questions related to the accuracy or integrity of any part of the work are appropriately investigated and resolved.

The study was approved by the research ethics committee of the Faculty of Medicine for Girls, Al-Azhar University, study ID 1639, number 2022121639. Informed consent and authorization were obtained from the parents or caregivers before enrolment in the study. Confidentiality of all data was ensured.

## Funding

None.

## Declaration of competing interest

The authors have no conflict of interest to declare.

## Data Availability

The data that support the findings are provided in the supplements.

## Appendix A Supplementary data

Supplementary material Image 1
