# Supplementary material for: Risk factors for autism spectrum disorder among infants and children admitted in a neonatal intensive care unit
Source: Dialogues Health. 2026 Mar 19;8:100288. doi: 10.1016/j.dialog.2026.100288 (PMC13054605; doi:10.1016/j.dialog.2026.100288)
Supplement: Supplementary file 1 — Supplementary material [file mmc1.docx]

**Supplementary Appendix**

**Supplement 1.** Causes of NICU admission

|  |  | **Non- autistic (n = 60)**  **No. (%)** | **Autistic**  **(n = 6)**  **No. (%)** | **P value** |
| --- | --- | --- | --- | --- |
| **Prematurity** | No | 55 (91.7) | 2 (33.3) | 0.002^*(1)^ |
|  | Yes | 5 (8.3%) | 4(66.7%) |  |
| **Intrauterine growth retardation** | No | 47(78.3%) | 6(100.0%) | 0.589^(1)^ |
|  | Yes | 13(21.7%) | 0(0.0%) |  |
| **Infant of diabetic mother** | No | 45(75.0%) | 6(100%) | 0.323^(1)^ |
|  | Yes | 15(25.0%) | 0(0.0%) |  |
| **Multiple pregnancy** | No | 52(86.7%) | 6 (100.0%) | 0.999^(1)^ |
|  | Yes | 8(13.3%) | 0(0.0%) |  |
| **Jaundice needs phototherapy** | No | 37(61.7%) | 4(66.7%) | 0.999^(1)^ |
|  | Yes | 23(38.3%) | 2 (33.3) |  |
| **Associated reproductive technique** | No | 52(86.7%) | 6 (100.0%) | 0.999^(1)^ |
|  | Yes | 8(13.3%) | 0(0.0%) |  |
| **Transient tachypnea of newborn** | No | 55(91.7%) | 6 (100.0%) | 0.999^(1)^ |
|  | Yes | 5(8.3%) | 0(0.0%) |  |
| **Pneumonia** | No | 57(95%) | 6 (100.0%) | 0.999^(1)^ |
|  | Yes | 3(5%) | 0(0.0%) |  |
| **Duration of NICU admission** | 7(5.75) | | 23(23.25) | 0.038^*(2)^ |

**(1) Fisher's exact test (2) Mann-Whitney U test *: Statistically significant at P ≤ 0.05**

**Supplement 2.** Patient characteristics in autistic and non-autistic cases

| **Patient characteristics** | | **Non- autistic**  **N(%)** | **Autistic**  **N(%)** | **P value** |
| --- | --- | --- | --- | --- |
|  | | 60(90.9%) | 6(9.1) |  |
| **Gender** | Female | 22 (36.7) | 2(33.3%) | 0.950^(1)^ |
|  | Male | 38(63.3) | 4(66.7) |  |
| **Age in months** | Median (IQR) | 25(22) | 6(13.75) | 0.158 ^(2)^ |
| **Gestational age (weeks)** | Median (IQR) | 37(0) | 32(7.25) | 0.032* ^(2)^ |
| **Weight for age** | **10th** | 2 (3.3%) | 1 ( 16.6%) | 0.156^(1)^ |
|  | **25th** | 10 ( 16.6%) | 1 ( 16.6%) |  |
|  | **50th** | 14 (23.3%) | 0 |  |
|  | **75th** | 8 (13.3%) | 2 (33.3%) |  |
|  | **90th** | 3 (5%) | 1 ( 16.6%) |  |
|  | **95th** | 3 (5%) | 0 |  |
|  | **Between 90th & 75th** | 3 ( 5%) | 0 |  |
|  | **Between 75th & 50th** | 5 ( 8.3%) | 0 |  |
|  | **Between 50th & 25** | 6 ( 10%) | 1 ( 16.6%) |  |
|  | **Between 25th & 10th** | 5 ( 8.3%) | 0 |  |
|  | **Between 10th & 5th** | 1 ( 1.6%) | 0 |  |
| **Length for age** | **Below 3rd** | 4 (6.6%) | 1 ( 16.6%) | 0.756^(1)^ |
|  | **5th** | 0 (0) | 0 |  |
|  | **10th** | 3 ( 5%) | 1 ( 16.6%) |  |
|  | **25th** | 11 ( 18.3%) | 0 |  |
|  | **50th** | 23 ( 38.3%) | 1 ( 16.6%) |  |
|  | **75th** | 7 ( 11.6%) | 1 ( 16.6%) |  |
|  | **90th** | 0 (0) | 0 |  |
|  | **95th** | 0 (0) | 0 |  |
|  | **97th** | 1 ( 1.6%) | 0 |  |
|  | **Between 90th& 75th** | 1 (1.6%) | 0 |  |
|  | **Between 75th&50th** | 3 ( 5%) | 1 ( 16.6%) |  |
|  | **Between 50th & 25th** | 7 ( 11.6%) | 0 |  |
|  | **Between 10th & 3rd** | 0 | 1 ( 16.6%) |  |

**(1) Fisher exact (2) Mann Whitney U test *: Statistically significant at P ≤ 0.05, IQR =Interquartile range**

**Supplement 3.** Maternal risk factors in autistic and non-autistic cases

|  | | | | **Non- autistic** | | **Autistic** | **P value** |
| --- | --- | --- | --- | --- | --- | --- | --- |
| **Maternal age** |  | | | 26.5±5 | | 26±3 | 0.801^(1)^ |
|  |  | | | N (%) | | N(%) |  |
| **Mode of delivery** | Normal vaginal delivery | | | 7(11.7) | | 1(16.7) | 0.55^(2)^ |
|  |  |  |  |  |  |  |  |
|  | Cesarean section | | | 53(88.3) | | 5(83.3) |  |
| **Diabetes Mellitus** | No | | | 45(75.0%) | | 6 (100.) | 0.32^(2)^ |
|  | Yes | | | 15(25) | | 0(0.0) |  |
| **Hypertension** | No | | | 52(86.7) | | 6 (100) | 0.97^(2)^ |
|  | Yes | | | 8(13.3) | | 0(0) |  |
| **Prolonged rupture of fetal membranes** | No | | | 59(98.3) | | 3(50) | 0.0017*^(2)^ |
|  | Yes | | | 1(1.7) | | 3(50) |  |
| **Antepartum hemorrhage** | No | | | 55(91.7) | | 5 (83.3) | 0.428^(2)^ |
|  | Yes | | | 5(8.3) | | 1(16.7) |  |
| **Meconium aspiration** | No | | | 57(95) | | 6 (100) | 0.999^(2)^ |
|  | Yes | | | 3(5) | | 0(0) |  |
| **Fever at any time of pregnancy** | No | | | 57(95) | | 5 (83.3) | 0.323^(2)^ |
|  | Yes | | | 3(5) | | 1(16.7) |  |
| **Type of feeding** | |  | **Autistic** | | **P value** | | |
| Breastfeeding | | N (%) | 2 (33.3) | | 0.831^(3)^ | | |
| Formula | | N(%) | 3(50) | |  |  |  |
| Mixed feeding | | N(%) | 1(16.7) | |  |  |  |

**(1) Independent T-test** **(2) Fisher Exact test** (3) **Monte Carlo test** ***: Statistically significant at P ≤ 0.05**

**Supplement 4. Auditory Brainstem Response (ABR) results of the right and left sides among the studied groups**

| **The Right / Left ear** | **Non- autistic** | **Autistic** | **P value** |
| --- | --- | --- | --- |
| **(ms)** | Mean± SD | Mean± SD |  |
| **Right wave I** | 1.89±0.3 | 1.73±0.1 | 0.259 |
| **Left wave I** | 1.9±0.19 | 1.9±0.4 | 0.691 |
| **Right Wave II** | 2.5±0.37 | 2.8±0.16 | 0.077 |
| **Left Wave II** | 2.6±0.3 | 3.2±0.72 | 0.130 |
| **Right Wave III** | 3.96±0.26 | 4.87±0.4 | 0.001 |
| **Left Wave III** | 4.04±0.38 | 5.2±0.5 | 0.001 |
| **Right Wave IV** | 5.1±0.37 | 5.58±0.24 | 0.002 |
| **Left Wave IV** | 5.05±0.35 | 6±0.4 | 0.001 |
| **Right Wave V** | 6.1±0.32 | 6.7±0.24 | 0.001 |
| **Left Wave V** | 6.1±0.4 | 7±0.2 | 0.001 |
| **Right, I-III interval** | 2.06±0.29 | 3.13±0.42 | 0.001 |
| **Left I-III interval** | 2.1±0.4 | 3.3±0.6 | 0.001 |
| **Right III-V** | 2.12±0.23 | 1.88±0.39 | 0.063 |
| **Left III-V** | 2.1±0.3 | 1.8±0.35 | 0.052 |
| **Right, I-V** | 4.2±0.27 | 5±0.33 | 0.001 |
| **Left I-V** | 4.2±0.37 | 5.1±0.3 | 0.001 |

Independent T-test

**Supplement 5.** Univariate logistic regression analysis to detect significant predictors of being autistic in ABR

|  | **Unadjusted odds ratio** | **95% CI** | **P value** |
| --- | --- | --- | --- |
| **Rt Wave III** | **552.98** | **5.058-60463.86** | **0.008*** |
| **Rt Wave V** | **4256.39** | **4.555-3977347.75** | **0.017*** |
| **Rt I-III interval** | **4478.18** | **0.88-22788231.18** | **0.054** |
| **Rt I-V** | **1602.722** | **5.045-509159.9** | **0.012*** |
| **Lt Wave III** | **63.038** | **2.997-1326.11** | **0.008*** |
| **Lt Wave V** | **242613.89** | **0.064-925138283864.144** | **0.109** |
| **Lt I-III interval** | **71.350** | **3.164-1608.767** | **0.007*** |
| **Lt I-V** | **2199.028** | **1.147-4216929.799** | **0.046*** |

***: significant at P ≤ 0.05 CI confidence interval**
